# Theta Oscillations Gate the Transmission of Reliable Sequences in the Medial Entorhinal Cortex

**DOI:** 10.1523/ENEURO.0059-20.2021

**Published:** 2021-05-27

**Authors:** Arun Neru, Collins Assisi

**Affiliations:** Division of Biology, Indian Institute of Science Education and Research, Pune 411008, India

**Keywords:** entorhinal, gating, grid cells, sequence, stability, theta

## Abstract

Stability and precision of sequential activity in the entorhinal cortex (EC) is crucial for encoding spatially guided behavior and memory. These sequences are driven by constantly evolving sensory inputs and persist despite a noisy background. In a realistic computational model of a medial EC (MEC) microcircuit, we show that intrinsic neuronal properties and network mechanisms interact with theta oscillations to generate reliable outputs. In our model, sensory inputs activate interneurons near their most excitable phase during each theta cycle. As the inputs change, different interneurons are recruited and postsynaptic stellate cells are released from inhibition. This causes a sequence of rebound spikes. The rebound time scale of stellate cells, because of an *h*–current, matches that of theta oscillations. This fortuitous similarity of time scales ensures that stellate spikes get relegated to the least excitable phase of theta and the network encodes the external drive but ignores recurrent excitation. In contrast, in the absence of theta, rebound spikes compete with external inputs and disrupt the sequence that follows. Further, the same mechanism where theta modulates the gain of incoming inputs, can be used to select between competing inputs to create transient functionally connected networks. Our results concur with experimental data that show, subduing theta oscillations disrupts the spatial periodicity of grid cell receptive fields. In the bat MEC where grid cell receptive fields persist even in the absence of continuous theta oscillations, we argue that other low frequency fluctuations play the role of theta.

## Significance Statement

The theta rhythm is a prominent oscillation in the brain and known to play a role in different forms of learning and memory, for its association with movement and its disruption in some pathologies. Oscillations in general, and theta in particular, are thought to coordinate the activity of distributed brain regions. Our study provides a mechanistic understanding of the role of theta oscillations in the generation of stable sequences of activity and its transmission across brain regions. We model a specific microcircuit (stellate cells coupled via inhibitory interneurons) based on the known architecture of the medial entorhinal cortex (MEC). This circuit motif occurs across various brain regions. Thus, mechanisms of spatiotemporal patterning observed in the MEC are recapitulated in other circuits as well.

## Introduction

The entorhinal cortex (EC) acts as a conduit between hippocampal and cortical circuits (Witter et al., 2017). Superficial layers of the EC receive multiple sensory inputs via the perirhinal and the postrhinal cortices and project to all hippocampal subfields (Cappaert et al., 2015). Given the diversity of inputs that arrive at the EC and its role as a hub, entorhinal networks must necessarily possess two attributes. They must represent an external input reliably and have mechanisms that allow them to flexibly select between competing inputs. Neurons in the medial EC (MEC) translate sensory input into temporally reliable and spatially confined representations (Hafting et al., 2005; Sargolini et al., 2006; Solstad et al., 2008). For example, grid cells in Layer II of the MEC fire at locations in space that form a striking hexagonally symmetric pattern (Hafting et al., 2005). The stability and precision of this pattern is remarkable given many experimentally measured variables in the MEC, inputs to stellate cells and local field potential oscillations, vary noisily as the animal navigates its environment. How can a stable spatial representation be built on such shaky ground? We discover that the answer lies in the interplay between theta oscillations, a characteristic slow rhythm present in many brain regions, and the intrinsic and network properties of the MEC. Disrupting theta by inactivating the medial septum, a prominent theta generator (Vertes and Kocsis, 1997), perturbs the spatially periodic receptive fields of grid cells (Koenig et al., 2011), and impairs the animal’s ability to navigate (Bolding et al., 2018). Each traversal over a particular region in space fails to generate a reliable response. The cumulative effect of this loss of reliability is that the crystalline grid-like structure of the neuron’s spatial receptive field dissipates into an amorphous pattern. In addition to generating a reliable representation, theta also plays a role in transiently coupling different brain regions to form functional networks (Benchenane et al., 2010; Takehara-Nishiuchi et al., 2012). This is evident in lateral EC (LEC), which, in contrast to the allocentric spatial response of the MEC, represents the location of objects in a particular context (Wilson et al., 2013) or encodes the passage of time between events (Tsao et al., 2018). The LEC forms part of a network including the medial prefrontal cortex and the hippocampus that are important in associative learning. Information transfer across the nodes of this network is gated by theta oscillations and the degree of association between different regions is reflected in the degree of theta synchrony between them (Takehara-Nishiuchi et al., 2012).

In a biophysically realistic computational model of an MEC microcircuit, we show that a stable grid pattern can be generated by coupling the network to theta oscillations from the medial septum (Vertes and Kocsis, 1997; Gonzalez-Sulser et al., 2014). Theta oscillations create periodic windows where the network is alternately receptive or resistant to perturbations. If cortical input arrives within the receptive window, it activates the corresponding interneurons. Competitive interactions between interneurons ensure that only those neurons receiving input are active while the others are inhibited. As the input changes, the locus of activity shifts. The rebound properties of stellate cells lead to spikes that mark the transition in activity from one interneuron to another. However, feedback excitation because of these spikes could trigger activity in postsynaptic interneurons that could compete with external inputs. The rebound time scale of stellate cells is such that these spikes are relegated to the least excitable phase of the interneurons. Theta oscillations cyclically order extrinsic inputs, inhibitory spikes by interneurons and rebound spikes by stellate cells. This ensures that the network listens only to extrinsic inputs and ignores its own activity. Further, we argue that the same mechanism, channeling relevant inputs only during the receptive phase of theta while relegating distractors to the resistant phases, is used by multiple brain regions to form transient functionally connected networks.

## Materials and Methods

### Neuron models

Stellate cells and interneurons were modeled as conductance based, single compartment spiking neuron models. In addition to sodium, potassium and leak channels, model stellate cells were also endowed with voltage dependent ion channels that were active at subthreshold membrane potentials. These ion channels enabled the neuron to produce sustained oscillations in response to a constant depolarization (Dickson et al., 2000; Acker et al., 2003; Rotstein et al., 2006). The model neurons share many of the theta frequency tuning properties observed in stellate cells. For example, when we drove the neuron using a linearly increasing frequency, we found that the amplitude of the oscillations was maximal for a frequency within the theta band. Further, we know that the slow time scale of the *h*–channel varies systematically along the dorsoventral axis of the MEC (Giocomo et al., 2007). In addition, the conductance of the *h*–current, the magnitudes of leak currents, persistent sodium currents, total amount of inhibition and unitary inhibitory currents are some of the other factors that vary along the dorsoventral axis of the MEC (Garden et al., 2008; Beed et al., 2013). A combination of these factors likely contributes to a systematic variation of the resonance frequency of our model neurons. Similar responses are seen in experimental recordings and model studies (see Giocomo et al., 2007; their Fig. 1; and Heys et al., 2010; their Fig. 4*D*).

The current balance equation for the equivalent circuit model of the membrane is given by the following:

Stellate cell,

(1) $$C\frac{dV}{dt}=I_{ext_{s}}-I_{Na}-I_{K}-I_{L}-I_{h}-I_{NaP}-I_{Syn}-I_{Noise};$$

Interneuron,

(2) $$C\frac{dV}{dt}=I_{ext_{i}}+I_{pulse}-I_{Na}-I_{K}-I_{L}-I_{Syn}-I_{Noise}-I_{\theta }.$$

Ionic currents were modeled as follows:

(3) $$I_{x}=g_{x}(v,t)(v-E_{x}),$$

where $g_{x}(v,t)=\text{ maximal conductance}\times f(\text{state of gating variables})$.

The gating variables followed first order kinetics. For example, the gating variable, *m* was given by the following:

(4) $$\frac{dm}{dt}=\frac{-(m-m_{\infty })}{\tau_{m}},$$

where both $m_{\infty }$ and *τ_m_* are functions of voltage.An equivalent representation is

(5) $$\frac{dm}{dt}=\alpha_{m}(1-m)-\beta_{m}m,$$

with $m_{\infty }=\alpha_{m}/(\alpha_{m}+\beta_{m})$; $\tau_{m}=1/(\alpha_{m}+\tau_{m})$.

The functional form of each ionic current and the gating variables, maximal conductances and reversal potentials are provided in Tables 1, 2.

**Table 1.** Functional form of conductances and gating variables

Ion channel	Current and its kinetics	Maximal conductance and reversal potential
Stellate cell Sodium	$\begin{array}{l} I_{Na}=g_{Na}m^{3}h(v-E_{Na})\\ \alpha_{m}=-0.1(v+23)/(e^{-0.1(v+23)}-1)\\ \beta_{m}=4e^{-(v+48)/18}\\ \alpha_{h}=0.07e^{(v+37.0)/20}\\ \beta_{h}=1/(e^{-0.1(v+7)}+1) \end{array}$	$\begin{array}{l} g_{Na}=52{\text{mS/cm}}^{2}\\ E_{Na}=55\text{mV} \end{array}$
Potassium	$\begin{array}{l} I_{K}=g_{K}n^{4}(v-E_{K})\\ \alpha_{n}=-0.01(v+27)/(e^{-0.1(v+27)}-1)\\ \beta_{n}=0.125e^{-(v+37)/80} \end{array}$	$\begin{array}{l} g_{K}=11{\text{mS/cm}}^{2}\\ E_{K}=-90\text{mV} \end{array}$
Persistent sodium	$\begin{array}{l} I_{NaP}=g_{NaP}ms(v-E_{Na})\\ \tau_{ms}=0.15\\ ms_{\infty }=1/(1+e^{-(v+38)/6.5}) \end{array}$	$\begin{array}{l} g_{NaP}=0.5{\text{mS/cm}}^{2} \end{array}$
HCN	$\begin{array}{l} I_{h}=g_{h}(0.65mhf+0.35mhs)(v-E_{h})\\ mhs_{\infty }=1/{\left(1+e^{(v+2.83)/15.9}\right)}^{58}\\ \tau_{mhs}=5.6/(e^{(v-1.7)/14}+e^{-(v+260)/43)})+1\\ mhf_{\infty }=1/(1+e^{(v+79.2)/9.78})\\ \tau_{mhf}=0.51/(e^{(v-1.7)/10}+e^{-(v+340)/52})+1 \end{array}$	$\begin{array}{l} g_{h}=1.5{\text{mS/cm}}^{2}\\ E_{h}=-20\text{mV} \end{array}$
Interneuron Sodium	$\begin{array}{l} I_{Na}=g_{Na}m^{3}h(v-E_{Na})\\ \alpha_{m}=0.1(v+35)/(1-e^{-(v+35)/10})\\ \beta_{m}=4e^{-(v+60)/18}\\ \alpha_{h}=0.07e^{(v+58)/20}\\ \beta_{h}=1/(e^{-0.1(v+28)}+1) \end{array}$	$\begin{array}{l} g_{Na}=35{\text{mS/cm}}^{2}\\ E_{Na}=55\text{mV} \end{array}$
Potassium	$\begin{array}{l} I_{K}=g_{K}n^{4}(v-E_{K})\\ \alpha_{n}=0.01(v+34)/(1-e^{-0.1(v+34)})\\ \beta_{n}=0.125e^{-(v+44)/80} \end{array}$	$\begin{array}{l} g_{K}=9{\text{mS/cm}}^{2}\\ E_{K}=-90\text{mV} \end{array}$

**Table 2.** List of default network and input parameters

Parameter	Symbol	Value
Synapse		
Maximal mutual inhibition conductance	*g_ii_*	1.0 mS/cm^2^
Maximal inhibitory conductance onto stellate cells	*g_ie_*	0.6 mS/cm^2^
Maximal excitatory conductance onto inhibitory interneurons	*g_ei_*	0.03 mS/cm^2^
Forward rate of inhibitory synapse	*α_s_*_,_*_inh_*	3.33 s^−1^
Reverse rate of inhibitory synapse	*β_s_*_,_*_inh_*	0.11 s^−1^
Forward rate of excitatory synapse	*α_s_*_,_*_exc_*	100.0 s^−1^
Reverse rate of excitatory synapse	*β_s_*_,_*_exc_*	0.33 s^−1^
Input		
Constant external current to stellate cells	$I_{ext_{s}}$	−2.7 μA/cm^2^
Constant external current onto interneurons	$I_{ext_{i}}$	0.2 μA/cm^2^
Baseline pulse current for interneuron	$p_{\vee ,i}$	−0.05 μA/cm^2^
Maximum pulse current for interneuron	$p_{\wedge ,i}$	1.0 μA/cm^2^
Amplitude of theta drive	*A*	0.04 mS/cm^2^
Threshold voltage for theta drive	*V_th_*	−80 mV

### Synapse model

Stellate cells were randomly connected to inhibitory interneurons with an excitatory synapse. Interneurons sent inhibitory connections to stellate cells as well as to other interneurons. The inhibitory population was modeled as an all-to-all connected network. The synapse was modeled as a conductance with a gating variable modulated by the presynaptic voltage.

(6) $$I_{syn}=g_{syn}s(v_{post}-E_{syn})$$

(7) $$\frac{ds}{dt}=F(v_{pre})\alpha_{s}(1-s)-\beta_{s}s,$$

where, $F(v_{pre})=(1+\tanh(v_{pre}/4))/2$, models the opening of a synaptic ion channel in response to action potential generated by the presynaptic neuron. The reversal potential determined the nature of synapse to be excitatory (when set to 0 mV), or inhibitory (when set to –80 mV).

### Connectivity

**Figure 1.**
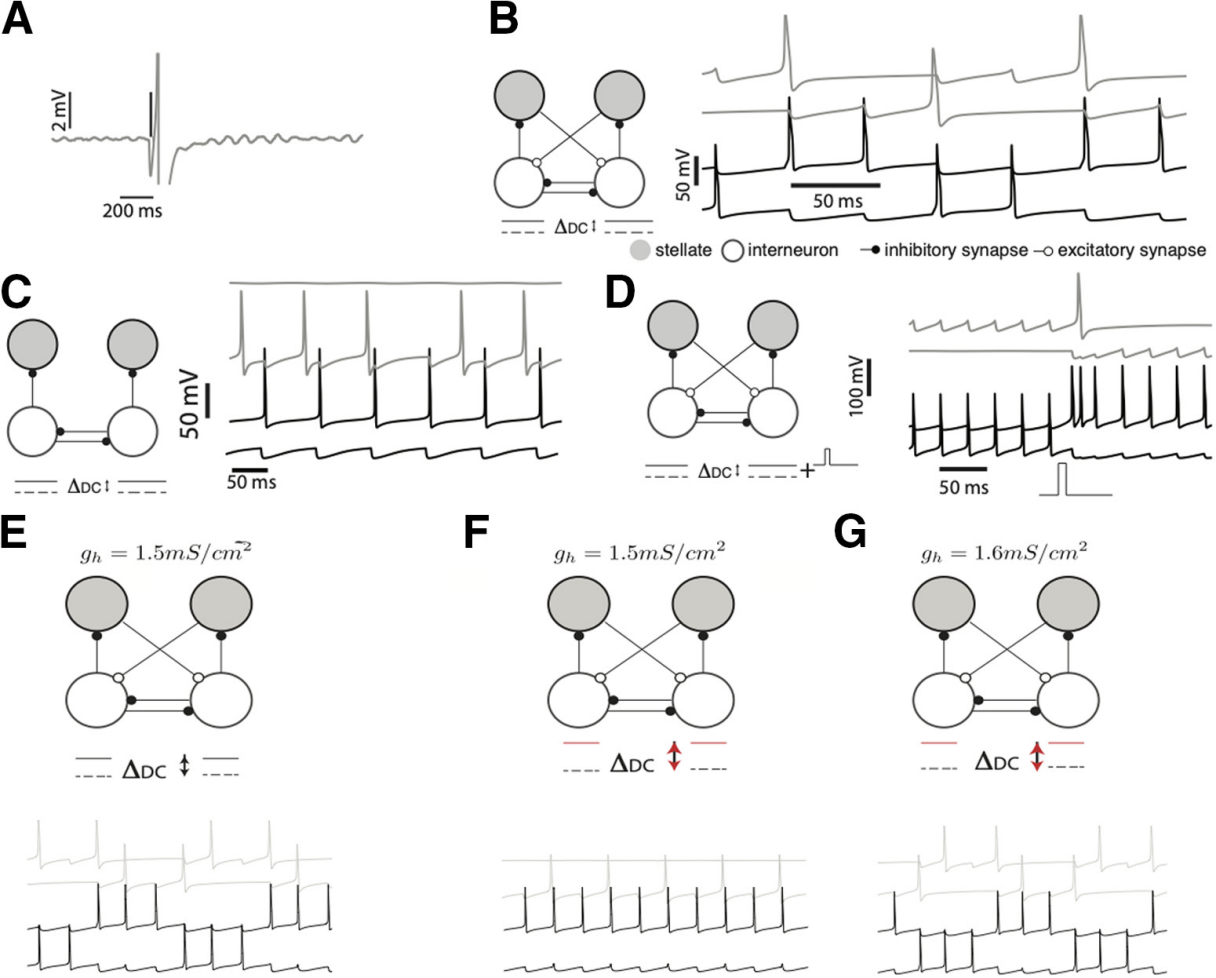
Dynamics of an MEC motif. Gray trace in ***A*** shows the rebound spiking response of a stellate cell to a single inhibitory spike. The time of the inhibitory spike is shown by the vertical line. In the absence of inhibition, the membrane potential of the stellate cell showed subthreshold oscillations. ***B***, A supra-threshold input, ΔDC ( DC–Direct Current; It is shown (solid) with reference to a baseline (dashed), The double-arrow shows the magnitude) applied to the inhibitory. Interneurons caused a rhythmic switching of activity in the network motif. Stellate cells spiked (gray traces) when the inhibitory interneuron (black traces) transitioned from activity to silence. ***C***, Switching failed in the absence of excitatory input; continuous firing of one interneuron (black trace) passively recruited the stellate cell (in gray trace), while the other interneuron (black trace) and corresponding stellate cell remained silent. ***D***, In a winner-take-all regime, both the interneurons received depolarizing inputs higher than that in ***B***. Only one interneuron fired continuously, a transient pulse toggled the activity of the inhibitory interneurons (black trace) and stellate cell spiked at the transition (top gray trace). ***E***, A network motif that operates as an autonomous oscillator. ***F***, Increased input to the interneurons transformed the system into a bistable switch. ***G***, Increasing the h-conductances transformed the system back into an autonomous oscillator.

The connectivity of the network motifs simulated here draws from several studies that have progressively established a detailed picture of the topology of the stellate cells and inhibitory interneuron networks (for review, see Witter et al., 2017). We modeled three network types. A small network motif comprised of two stellate cells and two interneurons (Fig. 1*B*), and two larger networks with stellate cells and interneurons arranged on rings. In one of the larger networks (Fig. 3*B*) with 40 stellate cells and 40 interneurons, the population of stellate cells and the population of interneurons were arranged on separate rings. Each interneuron sent projections to five adjacent stellate cells while each stellate cell sent projections to six randomly chosen inhibitory interneurons. In the network simulated in Figure 7, 80 interneurons were divided into two subpopulations 40 interneurons each. Each subpopulation connected to the common pool of stellate cells. The neighboring interneurons on each ring connected to neighboring stellate cells on the stellate cell ring. In all of the three network types we simulated, the interneurons were all-to-all connected.

### External input to the network

Theta rhythmic input to the network was provided by periodically modulating the excitability of interneurons using a sinusoidally varying conductance,

(8) $$I_{\theta }=A\sin(2\pi \omega t+\phi )(V-V_{th}),$$

where, *A* is the amplitude of theta rhythm; *ω* its frequency in Hertz. *V_th_* is the threshold voltage for the theta drive.In addition to a constant depolarizing input, a sequential pulse like input was used to drive the inhibitory interneurons. The temporal profile of the transient external pulse for an interneuron *i* is given by the following:

(9) $$I_{pulse}^{i}=\{\begin{array}{ll} p_{\vee }^{i} & \text{if }t<t_{s,min}^{i}\\ p_{\wedge }^{i}+(p_{\vee }^{i}-p_{\wedge }^{i})e^{(t-t_{s,k})/\tau_{r}} & \text{if }t_{s,k}^{i}<=t<t_{e,k}^{i}\\ p_{\wedge }^{i}+(p_{\vee }^{i}-p_{\wedge }^{i})e^{(t-t_{e,k})/\tau_{f}} & \text{if }t>t_{e,k}^{i} \end{array}.$$

The baseline value of the pulse was set to $p_{\vee }^{i}$. At time $t_{s,k}^{i}$, it rose to a maximum $p_{\wedge }^{i}$, with a rise time of *τ_r_*. At $t_{e,k}^{i}$, the pulse was switched off and fell to the baseline value with a fall time *τ_f_*. During a simulation, if the neuron received multiple pulses each was indexed by the variable *k*. Successive pulse-like inputs were then given to neighboring interneurons in successive theta cycles. The start time of a pulse to the *i^th^* neuron was calculated using the following prescription:

(10) $$t_{s}^{i}=\{t|t=iT+k\lambda ,\;k=0,1,....\}.$$

*T*, the time between pulses to successive interneurons, matched the period of the theta oscillation. The time between successive pulses to the same interneuron was given by *λ*. In the ring networks simulated here, the pulse visited all the interneurons before arriving back at the same neuron. Therefore, we set *λ* = *NT*. The duration of the pulse was set to *p_width_*. The end time,$t_{e,k}^{i}$, of a *k^th^* pulse to neuron *i* is

(11) $$t_{e,k}^{i}=t_{s,k}^{i}+p_{width}.$$

A similar sequence of pulses was given to the two rings of interneurons in Figure 8*A*. The top ring received inputs in a counterclockwise direction, while the order of inputs to the bottom ring followed a clockwise direction. In addition, all neurons were driven by a conductance-based noise drawn from a uniform distribution *U*(– 1,1). The reversal potential of the conductance was kept at –65 mV.

### Measure of reliability

To compare the reliability of the responses of the network for a given sequence of inputs across noisy trials, we used a measure of similarity between spike trains termed SPIKE distance (Kreuz et al., 2013). This measure can be calculated for each neuron across all pairs of trials and averaged over time. This value was calculated for a subset of neurons (*N *=* *8) that received input for all the theta frequencies that were simulated. The distribution of mean reliability across trials for each neuron is shown in Figure 5*C*. The analysis were implemented using a Python library, PySpike (Mulansky and Kreuz, 2016).

### Code accessibility

All the simulations were performed using a home-grown C++ library, *in silico*, that uses odeint, a boost C++ library to solve ordinary differential equations. The differential equations were integrated using Eulers method with a time step of 0.01 ms. Simulations were run on Linux servers with Intel Xeon E5-2670 processors running Ubuntu 18.04. The codes and the documentation required to run the simulations and analyze the outputs are available in the following GitHub repository linked here ( https://github.com/arunneru/theta_gates_reliable_sequences_mEC). The repository contains a Jupyter notebook that documents and implements all the steps required to simulate the codes and generate figures.

## Results

### Oscillatory and bistable dynamics of an MEC network motif

Layer II of the MEC consists of two distinct microcircuits with characteristic patterns of connectivity within and sparse connections across circuits (Witter et al., 2017; Nilssen et al., 2018). Stellate cells and fast-spiking parvalbumin positive (PV+) interneurons (Couey et al., 2013; Buetfering et al., 2014) form one circuit while pyramidal cells and 5HT3A interneurons form the other (Witter et al., 2017; Nilssen et al., 2018). This di-synaptic circuit motif, where principal neurons interact via an inhibitory intermediary, is prevalent throughout the EC (Couey et al., 2013; Fuchs et al., 2016; Nilssen et al., 2018). To understand how a network’s architecture affects its dynamics we simulated a simple network motif (Fig. 1*B*), a building block of the MEC, that consisted of biophysically detailed models of stellate cells (Dickson et al., 2000; Acker et al., 2003; Rotstein et al., 2006; Heys et al., 2010) and inhibitory interneurons (Wang and Buzsáki, 1996). Stellate cells generate characteristic subthreshold oscillations of their membrane potential in response to depolarizing inputs (Alonso and Klink, 1993). The frequency of subthreshold oscillations and the resonant frequency vary monotonically as a function of the magnitude and time scale of the *h*–current (*I_h_*; Garden et al., 2008; Giocomo and Hasselmo, 2008; Heys et al., 2010). Here, we modeled these properties using a hyperpolarization activated depolarizing current (*I_h_*; Dickson et al., 2000; Shay et al., 2016) and an amplifying persistent sodium current (*I_NaP_*; Magistretti and Alonso, 2002) in addition to leak and spiking currents (*I_L_*, *I_Na_*, and *I_K_*). We modeled interneurons using modified sodium and potassium currents that allowed them to spike at high frequency (Wang and Buzsáki, 1996). Both interneurons shown in Figure 1*B* received supra-threshold input. However, since they inhibit each other, only one of the neurons spiked while the other remained silent. Successive inhibitory spikes from an interneuron activated the depolarizing *I_h_* current in the postsynaptic stellate cell. This eventually drove the stellate cell to spike. Excitatory drive from the stellate cell activated the other interneuron of the pair that, in turn, silenced the first one. The activity of this motif switched rhythmically. The interneurons alternated between episodes of spiking and quiescence, and oscillated out of step with each other. Rebound spikes by stellate cells marked every transition from spiking to quiescence (Fig. 1*B*) and may serve as a viable mechanism to generate periodic firing fields of grid cells (Shay et al., 2016). Excitation because of rebound spiking caused rhythmic switching. When we removed excitatory inputs from stellate cells to the interneurons, only one interneuron remained active (Fig. 1*C*). Transitions in the activity of inhibitory interneurons can drive other stellate cells to fire, potentially leading to a pattern of activity where different neurons are sequentially activated. As the input to inhibitory interneurons increased so did its spiking frequency (Wang and Buzsáki, 1996). At higher frequencies, the time between inhibitory spikes was too short for the postsynaptic neuron to fire a rebound spike and cause a switch in the activity pattern (Fig. 1*D*). In this parameter regime, the network acted as a bistable switch where one of the interneurons remained active until a transient external perturbation toggled the switch (Fig. 1*D*). When inhibitory input to one of the stellate cells ceased, it emitted a rebound spike that marked the transition from one state of the network to the other (Fig. 1*D*). Thus, depending on the parameter regime, the motif simulated here can act as an autonomous oscillator (Fig. 1*B*) or as a switch (Fig. 1*D*) whose state could be toggled between activity and quiescence by a transient external perturbation. Further, we found that systematic changes to the *h*–channel conductance could predictably shift the system between a bistable and an autonomous oscillatory regime (Fig. 1*E–G*). Bistability is contingent on two key parameters in the model: the amplitude of depolarizing input to the interneurons and the synaptic strength. When we varied the input to the interneuron, we found that the bistable regime persisted for a broad swathe of inputs. Increasing the inhibitory synaptic conductance broadened the extent of the bistable regime. As the input to the inhibitory interneurons increased in magnitude, the frequency at which they fired also increased and tended to increase the threshold strength of an external input required to switch it from an active to an inactive state. We found that the dynamics of the motif simulated here, a building block of the MEC network, is stable to variation in some key model parameters. Despite our model’s concurrence with experiments and stability along some dimensions in parameter space, it is quite possible that slight changes in other parameters can lead it into a qualitatively distinct regime. Mittal and Narayanan (2018) have looked at precisely this aspect in the MEC. They simulated 150,000 variants of a stellate cell with diverse values of the channel parameters and found that 449 of these simulated neurons, scattered across a 55-dimensional parameter space, possessed electrophysiological responses that matched experimentally recorded stellate cells. Other neuron and network models also show similar variations as a function of key parameters (Prinz et al., 2004).

### Input-driven sequences in the MEC network

The EC receives sensory inputs from multiple cortical sources (Witter et al., 2017). Neurons in the deeper layers of the MEC, many of which are also grid cells, project to superficial layers, and innervate both the excitatory principal cells and inhibitory interneurons of Layer II. Almost half of these connections are targeted at the inhibitory interneurons (Ohara et al., 2018). Here, we examine the output of a model MEC network in response to a transient input that sequentially stimulates different interneurons in the network. Input was modeled as a brief pulse that lasted 40 ms; 85 ms later, we stimulated a different, randomly chosen interneuron. The onsets of successive pulses occurred 125 ms apart, the period of an 8-Hz theta oscillation.

**Figure 2.**
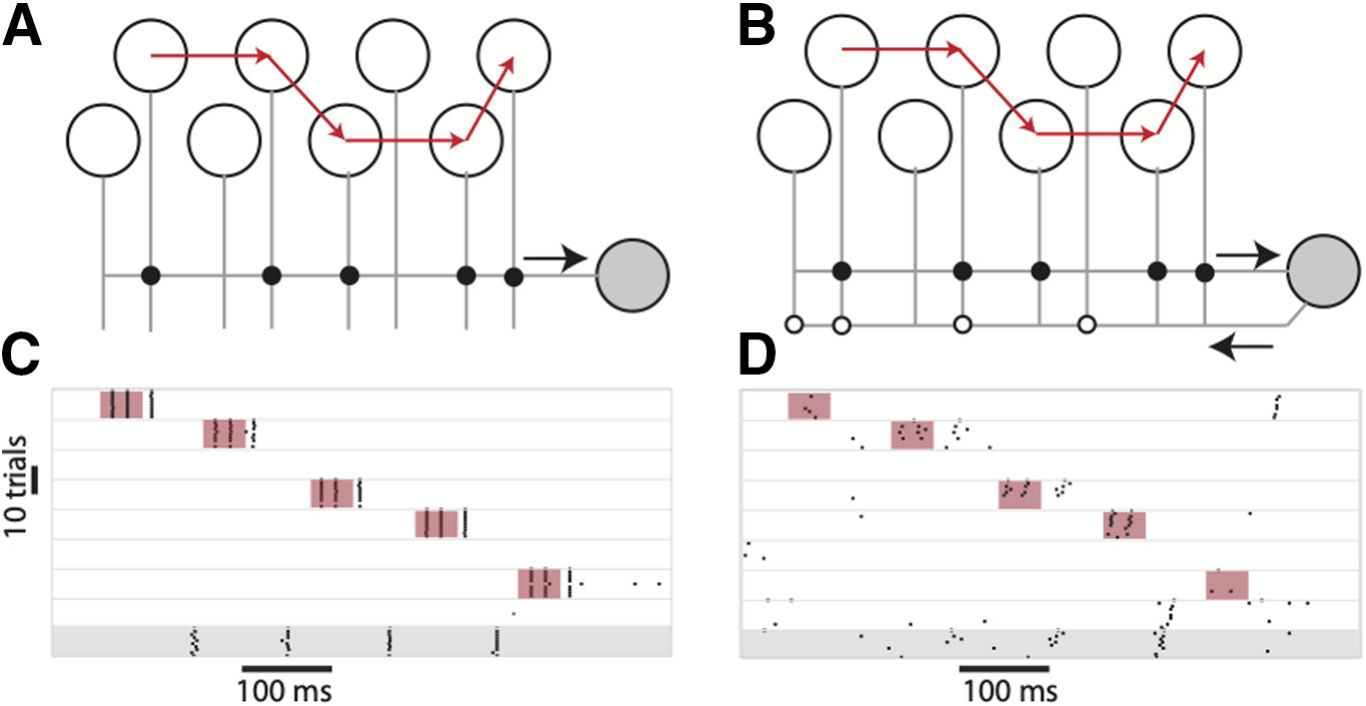
Stability of input driven sequences. ***A***, Network with sequential suprathreshold depolarizing pulses (temporal order of the input is shown by the red arrows) driving a subset of interneurons that were connected to a single postsynaptic stellate cell (filled gray circle). The response of the interneurons over 10 trials is shown as a raster plot in ***C***. The stellate cell (bottom raster shaded in gray) responded reliably over multiple trials. When feedback excitation from the stellate cell to a randomly selected subset of interneurons (***B***) was introduced, the response of the interneurons to the sequential input was perturbed and stellate cells did not spike reliably. The duration of the inputs is marked by the colored boxes in ***C***, ***D***.

In Figure 2*A*, all the inhibitory interneurons were connected to each other. The stellate cell received inputs from five randomly chosen interneurons. Our simulations compared two scenarios, one where there was no recurrent excitation from the stellate cells to the inhibitory interneurons (Fig. 2*A*) and another where each stellate cell randomly connected to six interneurons (Fig. 2*B*). In the absence of recurrent excitation from stellate cells, the interneuron that received external input generated spikes that inhibited all the other interneurons. When the pulse moved to a different interneuron, the locus of activity also shifted (Fig. 2*C*) and a rebound spike in postsynaptic stellate cells marked this shift. Since the stellate cell received inputs from multiple inhibitory interneurons, successive shifts from one interneuron to another elicited multiple rebound spikes from the stellate cell (Fig. 2*C*, bottom raster in gray background). Sequential activation of inhibitory interneurons and activation of stellate cells occurred reliably over multiple trials (Fig. 2*C*). Different trials were distinguished by continuous noise trains to stellate cells and interneurons with a mean amplitude that was 10% of the amplitude of the input to interneurons. When we introduced random excitatory connections from stellate cells to inhibitory interneurons (Fig. 2*B*), the response of the MEC network did not consistently follow the external drive (Fig. 2*D*). Interneurons received competing depolarizing inputs, one from the transient external drive and the other because of stellate cell spikes. The background noise and the history of activation determined which interneuron won this competition and silenced all others. This led to unreliable switching and considerable trial-trial variability in the activity of inhibitory interneurons. Therefore, the firing of stellate cells, that mark the shifts in interneuron activity, was also unreliable across trials (Fig. 2*D*, bottom trace in gray background).

### Theta oscillations reduce trial-trial variability of MEC responses

Feedback excitation from stellate cells stimulated postsynaptic interneurons in a manner that interfered with its response to an external input. Here, we sought to understand whether theta oscillations can attenuate the effects of excitation on the response of inhibitory interneurons.

#### Constraints on MEC network structure

**Figure 3.**
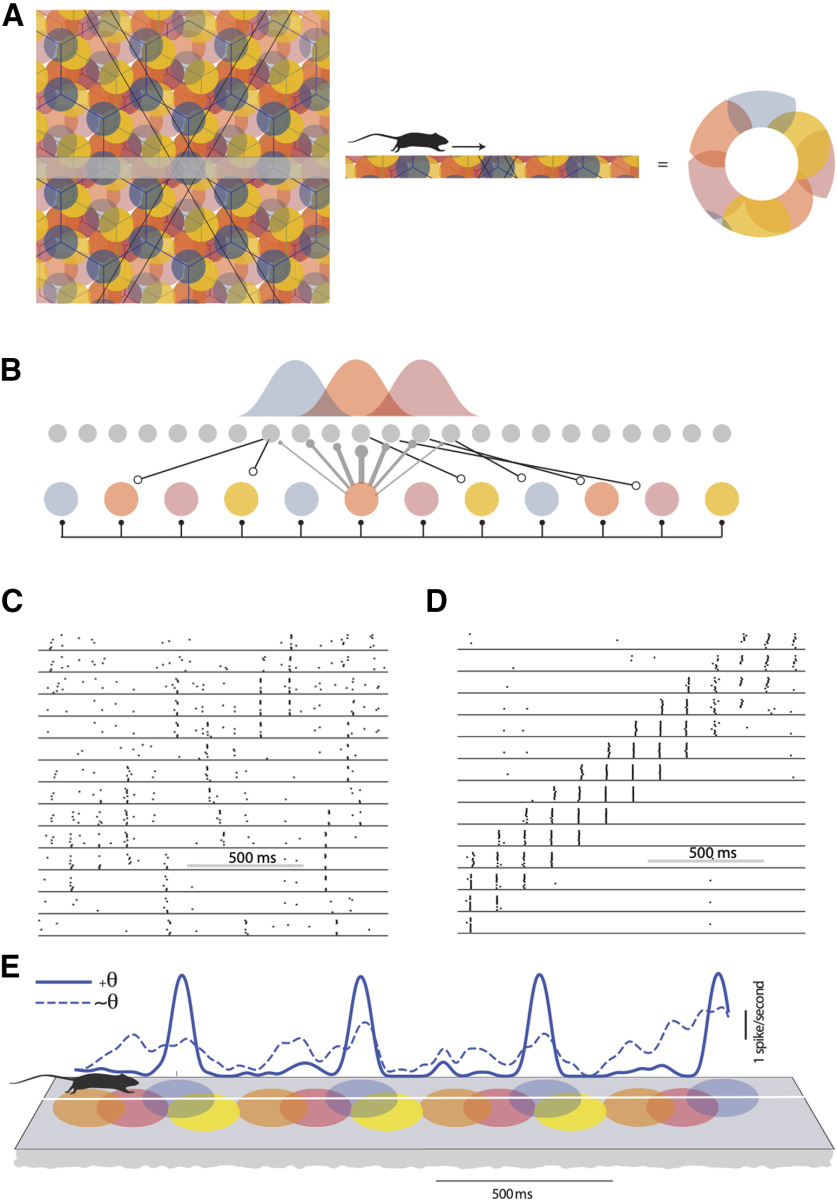
Theta mediated stability of an MEC network. A schematic representation of the overlapping grid-like receptive fields of four grid cells is shown in ***A***, left panel. As the rat traveled along a primary grid axis (marked in the figure as straight-line paths), the grid field repeated periodically (***A***, middle panel). This can be mapped to a circle (***A***, right panel). Stellate cells in the network (***B***, gray filled circles) received inhibitory input from the interneuron layer (colored circles). The strength of the inhibitory input followed a Gaussian profile (distribution shown in ***B***). Each stellate cell connected to a randomly selected group of interneurons. The response of the stellate cells in the absence of theta drive to the inhibitory interneuron layer is shown as a raster plot in ***C***. When theta oscillations (8 Hz) were present, stellate cells evoked a reliable response over ten trials (***D***). The mean firing rate convolved with a moving Gaussian window is shown in ***E***, when theta oscillations were present (solid line) or absent (dashed line). The schematic below shows the grid fields of a rat running at a uniform velocity. The temporally periodic spiking pattern of stellate cells appears as a spatially periodic response.

As in the previous section, we simulated a model MEC network that consisted of stellate cells and fast spiking inhibitory interneurons. In what follows, we describe some of the features of the responses of grid cells *in vivo* that provide clues to constrain the topology of the MEC network. The receptive fields of different grid cells form an overlapping patchwork that covers space (Hafting et al., 2005; Fig. 3*A*). Within a localized region of the MEC, grid cell receptive fields share the same spatial frequency and orientation, but are phase-shifted with respect to each other. We assumed, since stellate cells in our model were activated by inhibition, neurons with overlapping spatial receptive fields that can fire in close temporal proximity, and with similar response patterns, must also receive overlapping inhibitory input (Fig. 3*B*). What are the consequences of this assumption? If receptive field overlap is indeed a reflection of the overlap of input connections to stellate cells, it must also constrain the responses of the system in different, independent environments. Environmental and experiential changes to the receptive field of one grid cell must be correlated to similar changes in other cells such that the spatial phase relationships are preserved. Changes in distal environmental cues change the orientation of a grid cell’s receptive field. Changes in the shape of the enclosure can deform the receptive field (Krupic et al., 2015). As an animal becomes familiar with the environment, the scale of grid cell receptive fields shrink (Barry et al., 2012). Despite these transformations, the phase relationship between the receptive fields of two grid cells, remains invariant (Wernle et al., 2018), suggesting that the spatial contiguity of receptive fields must be a consequence of the topology of the underlying network. A recent study found a mapping between the spatial periodicity of the receptive fields of grid cells and their physical location in the MEC (Gu et al., 2018). Grid cells with similar phases were arranged close to each other. This phase pattern repeated anatomically in a lattice that resembled the grid-like receptive fields of individual neurons. Further, the direction of movement of the animal was accompanied by a directed spread of grid cell activity in a local anatomic neighborhood. A similar topographic mapping between the behavioral states (head direction) and neuronal network structure (ring attractor) is found in the *Drosophila* central complex (Seelig and Jayaraman, 2015). More recently, the activity of the mammalian head direction circuit has been mapped to a low dimensional ring attractor (Chaudhuri et al., 2019). These observations are consistent with the architecture of attractor models, a class of theoretical models used to explain how grid cell receptive fields emerge from a network of neurons. Attractor networks typically possess a periodicity (or symmetry) that allows the component neurons to fire in a spatially periodic manner. For example, periodic activity may arise because of the radial symmetry of the connectivity kernel that leads to a hexagonally symmetric stable solution (Burak and Fiete, 2009). In other models (Guanella et al., 2007; Navratilova et al., 2012), the periodic structure of network leads to periodic patterns of activity.

Figure 3*A* shows the overlapping receptive fields of four stellate cells. The receptive fields have the same spatial period, but are phase-shifted with respect to each other. From the responses of these four neurons, one cannot identify a unique point where the animal would be located. The locus of points where the activity of the neurons is identical lie at the vertices of a triangular grid. Given the symmetry of grid cell responses, all equivalent locations in space can be mapped to a single point on a torus (Shilnikov and Maurer, 2016). Any straight-line trajectory along a primary grid axis generates a periodically repeating pattern of activity that can be mapped to a circle (Yoon et al., 2016; Fig. 3*A*). In keeping with our assumption that overlapping responses are generated by stellate cells with overlapping inputs, we modeled the stellate-interneuron network as a ring where “neighboring” stellate cells that fire in close spatial and temporal proximity received inputs from overlapping groups of interneurons (Fig. 3*B*). The strength of synaptic input from an interneuron to the stellate cell layer followed a Gaussian profile. The interneurons were all–to–all connected in this network (Fig. 3*B*). The neighborhood relationship between interneurons was therefore inherited from the inputs that drove the interneurons and their connections onto the stellate cell layer. In contrast to the structured and local connectivity from interneurons to stellate cells, feedback excitation onto the inhibitory layer was random. We modeled the trajectory of the animal by sequentially activating neighboring inhibitory interneurons with a transient input. In our simulations, we assumed that inhibitory interneurons received the same input every time the animal was at a particular location. Each vertex of the hexagonal receptive field of a particular grid cell showed the summed spiking activity of the neuron at that location. Therefore, the ability to reliably follow the input (how the cell responded in time) translated to the ability to respond in a grid like pattern. The raster plots (Fig. 3*C*) show the response of a subset of 20 stellate cells to a temporally varying input. Predictably, the response of the network was not reliable across trials as it did not always follow the input. The activity of one of the neurons, averaged across 10 trials, is shown in Figure 3*E*. Because of the ring like topology of the network, the neuron periodically received a supra-threshold input. The neuron was just as likely to fire when an input was present as when it was absent (Fig. 3*E*, dashed line).

#### Inputs to MEC are modulated by theta oscillations

Reliable responses of principal neurons in the EC and the hippocampus are often contingent on the presence of theta. Some spatial inputs come packaged in bursts locked to theta oscillations. One source of theta to the MEC is a central pattern generator in the medial septum (Vertes and Kocsis, 1997) that extends GABAergic connections to the inhibitory interneurons and periodically modulates their firing rate (Gonzalez-Sulser et al., 2014). GABAergic orchid neurons in the medial septum fire bursts of spikes in each theta cycle (Viney et al., 2018). Head direction cells in the MEC are typically clustered in the deeper layers and send inputs to patches in Layer II where grid cells are found. These neurons also fire spikes in a theta cycle-by-cycle manner (Burgalossi et al., 2011; Brandon et al., 2013). Further, the phase of stellate cell spikes are consistently shifted with respect to head direction cells in each theta cycle (Brandon et al., 2013). Pyramidal cells extensively innervate all neuron types of the MEC except head direction cells. Brief optogenetic activation of pyramidal cells during each theta cycle disrupts coherent firing of grid cells at the vertices of a hexagonal pattern only within a small window of the theta cycle. The system regains its ability to fire precisely at grid fields in the remaining window of the theta cycle (Zutshi et al., 2018). This observation suggests that path-integrated inputs essential for reliable grid formation arrive as afferent input to the grid cell network. Otherwise, the effect of disrupting path integration in one cycle would cascade over successive cycles. Head direction cells remain untouched when pyramidal neurons are optogenetically activated (Zutshi et al., 2018) and form an important component of the path integration signal. Further, changes in speed lead to predictable changes in medial septum theta oscillations. Therefore, inputs required for path integration, namely, speed and direction, are, at least partially, represented in theta frequency (Jeewajee et al., 2008) and input from head direction cells (Taube et al., 1990a,b), respectively. A significant part of this input drives inhibitory interneurons in a theta cycle dependent manner.

We implemented theta rhythmic modulation of the MEC network by periodically (6–12Hz) driving the entire population of inhibitory interneurons. The pulse like input that was used to drive the neurons remained the same as in earlier simulations (Fig. 2; Materials and Methods). Neighboring interneurons were recruited in successive cycles of the theta oscillations (125 ms apart). As neighboring inhibitory interneurons provided input to overlapping groups of stellate cells, the same stellate cell was repeatedly released from inhibition and in successive cycles. A combination of conductances in stellate cells endowed them with the ability to generate rebound spikes when inhibitory input abruptly ceased (Ferrante et al., 2017). Our model shows, as others have (Shay et al., 2016), that phasic inhibitory inputs can depolarize stellate cells. We found that the presence of theta oscillations ensured that only those neurons receiving an external input fired and suppressed the activity of all the other interneurons. The output generated in response to a sequential input pattern was stable across noise trials (Fig. 3*D*). The firing rate map (Fig. 3*E*, solid line) calculated for one neuron as an animal traversed a linear track (Fig. 3*E*, schematic) is shown. Assuming that the animal moved at a uniform velocity, it encountered each grid field (alternately, received an external input) after a fixed interval of time and reliably generated a sequence of spikes in response to the input. Note that uniform velocity is not a prerequisite for stable stellate cell responses. Changes in velocity are accompanied by changes in the frequency of theta oscillations (Jeewajee et al., 2008). Our simulations show that stable sequences are generated despite dynamic changes in the frequency of theta that can speed up or slow the onset of activity in successive neurons (Fig. 5*D*).

Our model network consisted of an equal number of stellate cells and inhibitory interneurons. This is in contrast to Layer II of the MEC where stellate cells are known to vastly outnumber interneurons. However, this does not pose a problem for our model, provided the distribution of inhibitory inputs to stellate cells is maintained. The stellate cells and inhibitory interneurons are organized in a ring network. Neighboring stellate cells received inputs from overlapping sets of inhibitory interneurons. Therefore, neighboring stellate cells also possess highly overlapping receptive fields. Adding more stellate cells in our model would result in denser overlap of receptive fields. This is evident in responses seen in the raster plot shown in Figure 3*D*.

### Mechanism of theta-induced reliability

**Figure 4.**
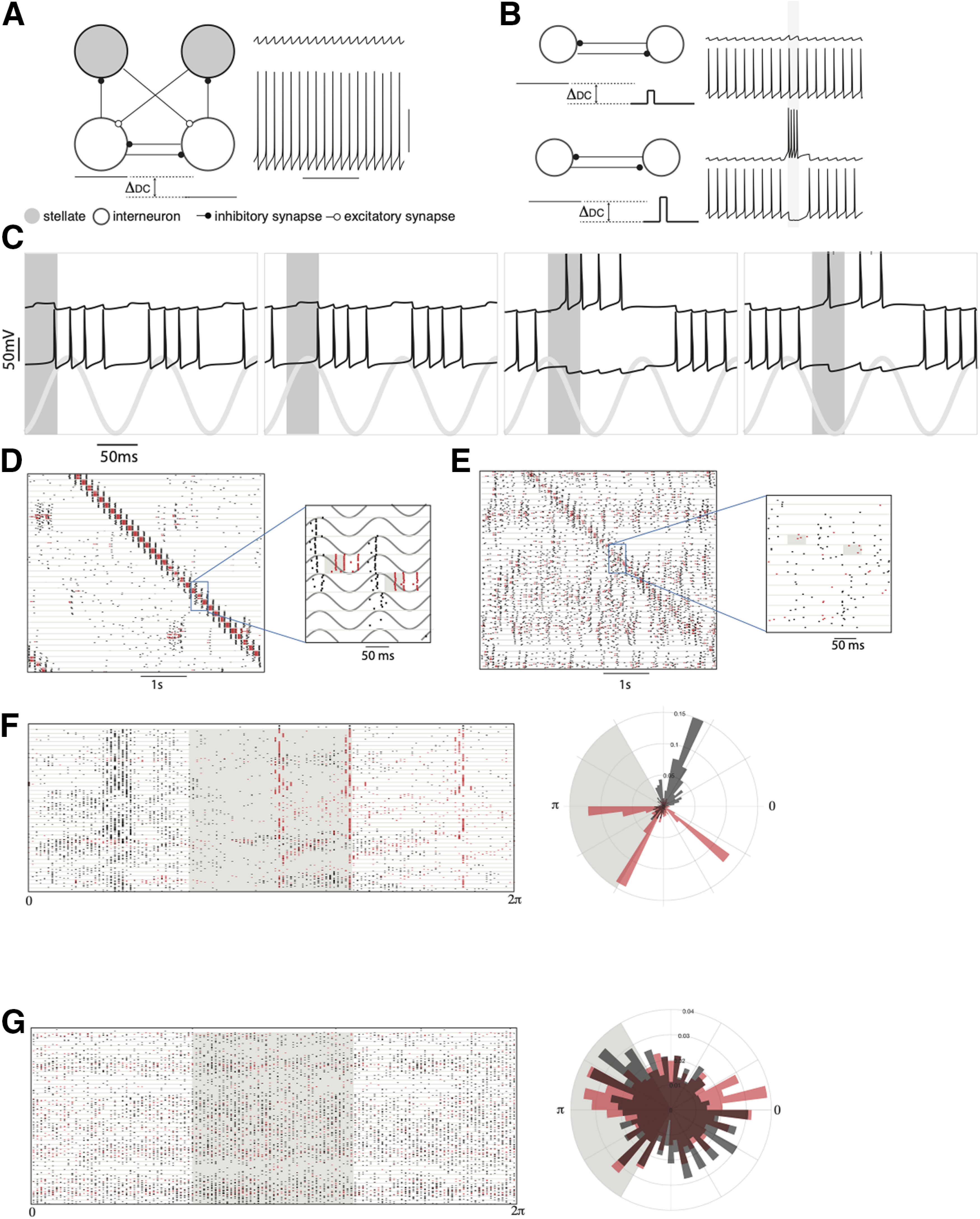
Mechanism of theta-induced reliability. ***A***, Membrane potential of inhibitory interneurons when they received different suprathreshold inputs. The neuron receiving the higher input fired continually (bottom trace) while the other remained silent (top trace). ***B***, The same network motif as (***A***; stellate cells not shown) was simulated. A weak transient pulse was given to the interneuron receiving the lower DC (Direct current) input (***B***, top panel). The stimulated interneuron (top trace) did not switch. A strong pulse caused a successful switch (bottom two traces). ***C***, Theta rhythmic drive was given to both the interneurons. A weak pulse (duration of the pulse is marked by the gray bar) caused a switch when it occurred in some phases of the theta oscillation (bottom gray trace) but not during others. ***D***, Raster plot showing the reliable response of a subset of 20 interneurons (red line) and 20 stellate cells (black lines) from a larger network of 80 neurons, to an external input (marked as a gray bar in the magnified raster plots). The response of the same network in the absence of theta oscillations is shown in ***E***. Each row in ***D***, ***E*** shows the response of a neuron across 10 trials which are plotted between the horizontal lines in the raster plots. ***F***, left, Raster plot shows the phase (with respect to the theta oscillation) at which stellate cells (black lines) and interneurons (red lines) spiked. The gray area marks the phase of the oscillation when the stimulus was present. Right, Polar plot showing a histogram of the phases where spikes occurred. The external input is marked in gray. ***G***, Response of the network when theta oscillations were absent. The phase was defined in terms of the input pulse that sequentially and periodically stimulated neighboring interneurons.

How do theta oscillations affect the network such that it responds selectively to an external input and not to distractors, namely, competing excitatory spikes from stellate cells? The networks simulated in Figure 3 operated in a regime where a transient drive or an excitatory spike can cause a perturbation that toggles the activity of the interneurons (Fig. 1*C*). To understand the effect of theta oscillations on the network, we first simulated a simple network (Fig. 4*A*) where the two interneurons received different constant depolarizing inputs. The neuron receiving the higher input continually spiked while the other neuron was quiescent. In order to toggle the activity of this network, we stimulated the quiescent neuron with a transient pulse. For sufficiently strong pulses, the neuron’s activity switched for the duration of the input (Fig. 4*B*, bottom traces). Weaker pulses, on the other hand, did not evoke a transient switching response (Fig. 4*B*, top traces). Here, the network motif was simulated in the bistable regime (Fig. 1*D*). Stellate cells can also generate autonomous periodic spiking (for a detailed phase response analysis, see Acker et al., 2003). Next, we stimulated the interneurons with a periodic theta drive that modulated the firing rate of the interneurons. Interneurons were maximally depolarized at the trough of theta and were most likely to spike then. When a weak pulse arrived at the quiescent neuron during the depolarizing phase of the theta oscillation, it successfully toggled the network. However, inputs that arrived at other phases did not cause a switch (Fig. 4*C*). Therefore, theta created periodic temporal windows where the activity of the network could be switched from one interneuron to another. In a larger network, we found that when the input arrived at the receptive phases of theta, the stimulated interneurons responded reliably to the input and the locus of activity of the network followed the input (Fig. 4*D*). However, merely creating periodic windows where external inputs can drive the network is not sufficient to generate reliable activity. Excitatory spikes from stellate cells can also occur during this receptive phase and perturb the response of the network to an external input. We found that when theta oscillations were present, the stellate cell spikes were tightly synchronized (Fig. 4*D*). These spikes always followed a burst of activity in the presynaptic interneurons (Fig. 4*D*, inset, *F*). The interneurons were locked to the trough of theta oscillations while stellate cell spikes occurred at a later phase because of the time taken to generate a rebound spike when released from inhibition. The stellate cell spikes occurred when the interneurons were hyperpolarized and were impervious to feedback excitation from stellate cells (Fig. 4*D*,*F*). The input-triggered response of the network segregated inhibitory interneuron spikes from the stellate cell responses (Fig. 4*D*). When theta was present and the external input occurred at a receptive phase, it was followed by a reliable burst of interneuron spikes, followed by the activity of stellate cells. In the absence of theta, stellate cells fired after each burst of interneuron spikes (Fig. 4*E*, inset). However, the stellate cell spikes were broadly distributed throughout the time between successive inputs to the network (Fig. 4*E*,*G*). Thus, in the absence of theta, stellate cells could effectively perturb the activity of the network to act as a “distractor” that derailed network’s response to an external drive. Earlier studies have looked at the oscillatory mechanisms that separate target network responses from distractors (for review, see Lengyel et al., 2005). For example, Hopfield (1995) used oscillations to create concentration-invariant odor representations that could be read out by follower networks using time-delay lines to separate the target pattern from distractors. Work by Li and Dayan (1999) consider the case of pattern recognition by selective amplification. They showed that excitatory-inhibitory networks tended to fall into spurious attractors termed “hallucinations.” The ability of the system to elude these spurious attractors improved when the network autonomously generated oscillations. These studies, like ours, use oscillations to construct noise-free representations. However, the mechanism we employ is different from that used in earlier studies. We show that inputs that arrive within a window of theta oscillations can elicit a response from stellate cells, while those that do not arrive within the receptive phase window, have a higher threshold to elicit a similar response. Distractors (recurrent excitation within the MEC network) get relegated to the hyperpolarized phase of the theta oscillations. This temporal organization of stellate cell and inhibitory interneuron spikes with respect to a theta clock ensure that the network listens to external inputs while ignoring its own recurrent excitatory activity.

### Theta-induced reliability persist despite qualitative changes in topology of feedback excitation

**Figure 5.**
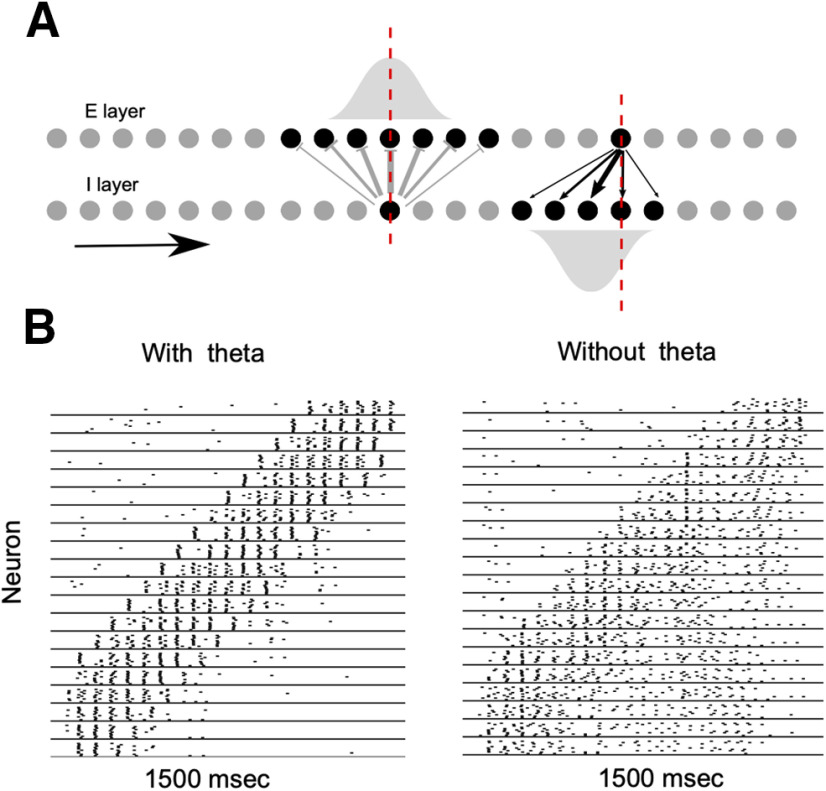
Theta-induced reliability persists despite qualitative changes in topology of feedback excitation. ***A***, Network topology. The stellate cells are arranged in the top layer and interneurons in the bottom layer. Each interneuron projects to its postsynaptic stellate cells with the weights that follow a Gaussian profile. Each single stellate cell projects to local postsynaptic interneurons with a Gaussian connectivity profile instead of random connections used in the previous simulations (shown in Fig. 3*B*). The Gaussian connectivity profile of the stellate neuron was asymmetrically shifted with respect to the central position occupied by the neuron in the ring. ***B***, Raster plot showing the response of a subset of neurons across trials in the presence (left) and absence (right) of theta rhythmic input to interneurons.

Stellate cells excite inhibitory interneurons (Couey et al., 2013; Buetfering et al., 2014). We modeled feedback excitation as random inputs from stellate cells to the inhibitory layer. Here, we ask the following: are the disruptive effects of feedback excitation a consequence of the the specific network topology? To address this question, we simulated a network of stellate cells and interneurons arranged on a ring as we did in previous simulations. However, instead of introducing broad random feedback excitation to the inhibitory interneurons, we chose local asymmetric inputs from each stellate cell (Fig. 5*A*). Figure 5*B* shows the response of the network to sequential input. In the absence of theta rhythmic drive, the network activity reflected influences from both the sequential input pattern and the intrinsic dynamics of the network. The asymmetry of connections dictated the direction along which the activity of the network spread. However, it did not reliably represent the input. In contrast, when theta oscillations were present, and the inputs arrived in a theta phase-locked manner, the output of the network faithfully followed the input (Fig. 5*B*, left panel). We show, regardless of the specific form of recurrent excitation, patterns of activity were disrupted when theta oscillations were absent. Therefore, recurrent excitation perturbs the response of the network to dynamic input and its effects are not an artifact of the specific connectivity profile.

### Theta-induced reliability persists over a range of frequencies

**Figure 6.**
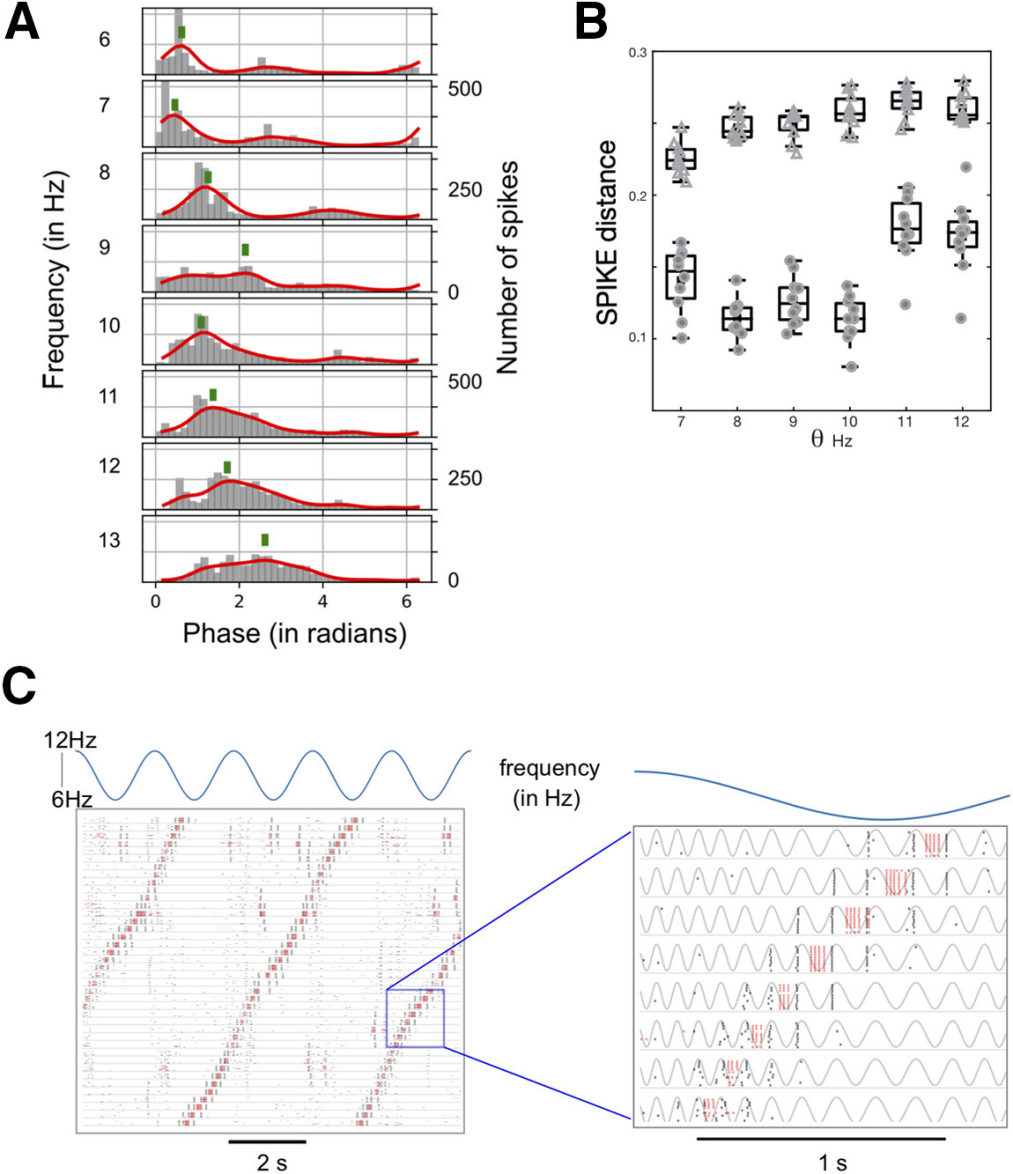
Reliability across a range of theta frequencies. ***A***, Histogram showing the distribution of phases of stellate cell spikes for a range of theta frequencies from 6 to 15 Hz. The green marker locates the mean of the distribution (***B***) box plot of the SPIKE distance (Kreuz et al., 2013) in the presence (filled circles) and absence (triangles) of theta as a function of the rate at which input pulses stimulated successive interneurons. ***C***, Raster plot showing the response of the neurons (40 stellate cells, black lines; 40 interneurons, red lines) across ten trials (as in Figs. 4*D*,*E*, 5*B*). The instantaneous frequency of the theta oscillation was varied from 6.0 to 12.0 Hz in a periodic manner (blue trace shows the instantaneous frequency of the theta oscillation). A magnified version of the raster plot on the left shows spikes against a background of theta oscillation. Notice the theta frequency decreases in a manner indicated by the blue trace above.

Theta oscillations span a range of frequencies from 6 to 12 Hz in rodents. Do changes in theta frequency compromise the reliability of the network responses? As theta frequency increased, the time between inhibitory bursts and rebound spikes remained nearly the same since this was determined by the time scale of the rebound kinetics of stellate cells. Therefore, the phase at which stellate cell spikes occurred started to shift systematically as the frequency increased (Fig. 6*A*). As the distribution shifted rightward (Fig. 6*A*), it became more likely that stellate cell spikes would invade the receptive phases of the theta oscillation (Fig. 4*C*) and interfere with the ability of the network to follow an external input. We calculated the reliability across trials measured as the pairwise dissimilarity between spike trains (see Materials and Methods). This measure showed greater reliability (Fig. 6*B*, lower spike distance, circles) for theta between 6 and 12 Hz compared with a network that was not driven by theta oscillations (Fig. 6*B*, triangles in the box plot). Here, we simulated the network for a particular value of the time scale of the *h*–current, *τ*, that varies along the dorsoventral axis of the MEC. Given the broad range of theta over which the network remains reliable, we anticipate that other values of *τ* would also give us a reliable response. However, dorsoventral gradients in *τ* can shift the regime where the network is reliable. Changes in theta frequency can signal changes in the animal’s behavior. For example, theta frequency is linearly related to the animal’s velocity (Jeewajee et al., 2008) and can change dynamically with changes in velocity. As the animal’s velocity increased, it encountered grid fields more rapidly. This translated in our model as an increased rate at which external pulses arrived at neighboring interneurons. We found that the network responded reliably to external inputs despite velocity dependent changes in frequency (Fig. 6*C*).

### Theta oscillations gate the transmission of competing inputs

**Figure 7.**
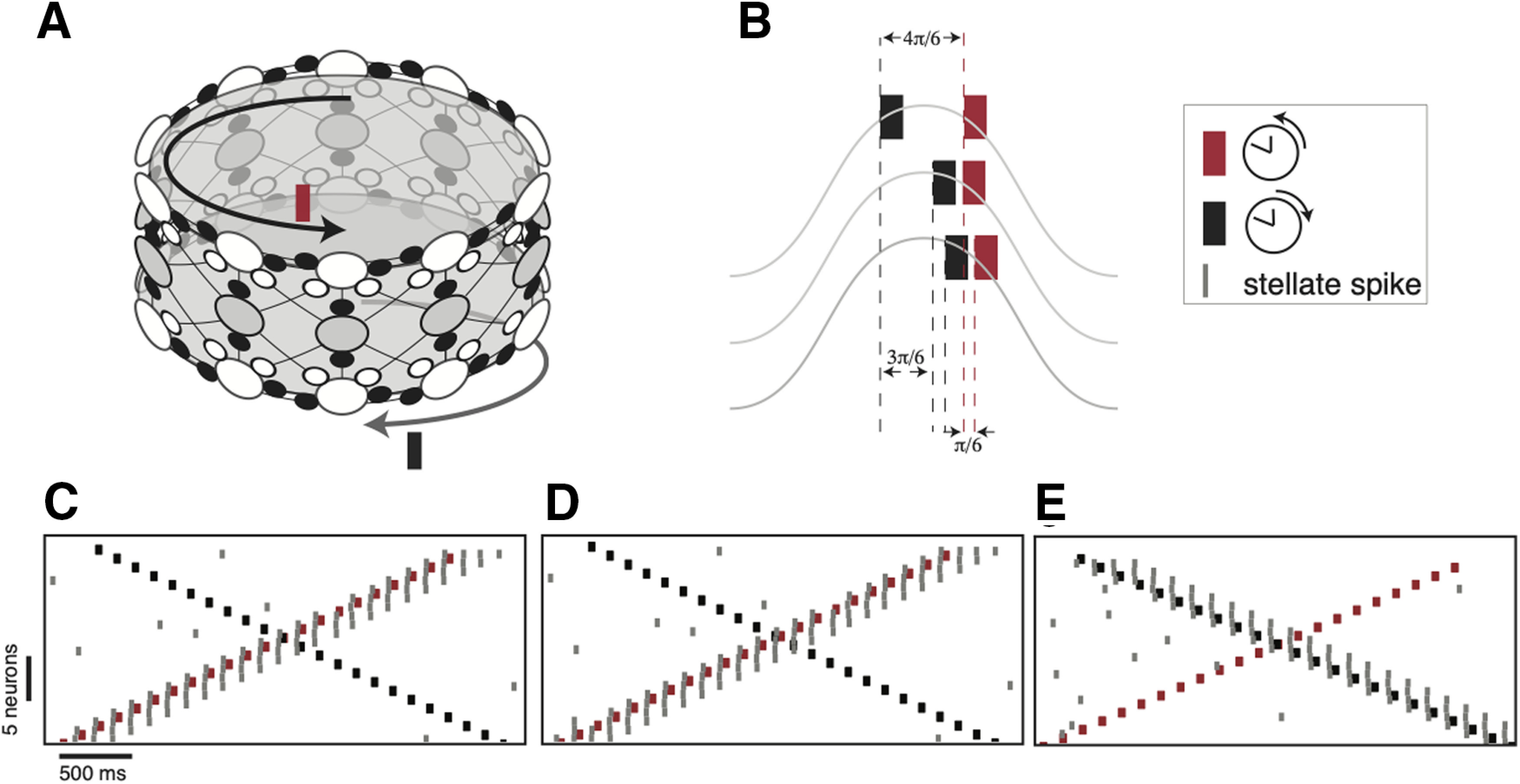
Theta gates transmission of competing inputs. ***A***, Topology of the network. Two sets of interneurons arranged on different rings (top and bottom empty circles) inhibit the same stellate cell population (middle ring with filled circles). Two separate input pulse trains (red and green bars) are given to the two inhibitory population. The input to upper ring followed a counterclockwise activity pattern while input to the lower ring followed a clockwise sequence. ***B***, The phase of each input within a single cycle of theta for three different cases. The response of the network to these patterns of input are shown in ***C–E***. The peak of the oscillation corresponds to the maximally hyperpolarized phase of theta. ***C***, The input arriving at the depolarizing phase (red) elicited a spike (gray line) in the postsynaptic stellate cell while the one arriving during the hyperpolarized phase elicited none (this case corresponds to the top trace in ***B***). ***D***, Both the inputs arrive at the depolarizing phase. The input that caused a maximum depolarization (red) elicited a successful response (this case corresponds to the middle trace in ***B***). ***E***, Inputs to both the clockwise and the counterclockwise rings were shifted. This switched the stellate cell from following the counterclockwise input to following the clockwise input.

When theta oscillations were present the network reliably followed external inputs that arrived at the receptive phase of theta. Here, we examine the response of the network when competing external inputs occur at different phases of the theta cycle. We simulated a network consisting of two groups of interneurons connected to the same pool of stellate cells. All the interneurons were coupled to each other. Each group of interneurons extended inhibitory connections to the stellate cells forming two separate ring networks (Fig. 7*A*). The upper ring of interneurons received a transient pulse that traveled in a counterclockwise direction while the lower ring received an input that traveled clockwise. The two rings were chosen to distinguish different input streams. The clockwise and counterclockwise streams competed to elicit a response in the same pool of stellate cells. We kept the amplitude of both the inputs constant and varied their phase relationship with respect to an external common theta oscillation.

In the first of the cases tested (Fig. 7*B*), one of the inputs (clockwise) arrived when the interneurons were near their most hyperpolarized phase while the second input (counterclockwise) arrived at the depolarizing phase. Predictably, the second input succeeded in eliciting a sequence of spikes in the upper ring of interneurons that entrained the stellate cells and inhibited all the other neurons. We then varied the phase of the clockwise input such that the pulse occurred progressively closer to the phase of the counterclockwise input (Fig. 7*B*,*D*,*E*). When the input crossed a particular phase of the theta oscillation (Fig. 7*B*, bottom row, *E*), it elicited spikes in inhibitory interneurons of the lower ring. This inhibited all the other interneurons including the neurons on the upper ring of the network. Therefore, although the counterclockwise inputs occurred during a depolarized phase of theta, the response of the network followed the clockwise inputs because it occurred earlier and also during the depolarized phase of the theta cycle. Thus, the mechanism whereby the network selects between inputs is determined, not only by coherent theta gain modulation of inputs, but also by the temporal order and the phase at which the inputs occur. Thus, theta oscillations can serve as a gate that permits a particular temporal ordering of stellate cell responses while prohibiting a different temporal ordering in the same group of cells.

Several studies have shown that theta oscillation coherence plays an important role in interregional interactions. However, few studies have looked at how this can be implemented in different brain networks. Akam and Kullmann (2010) was the first study that provided a mechanistic understanding of how oscillations can be used to separate different streams of inputs in brain networks (termed demultiplexing). The form of demultiplexing implemented in our model is termed time division multiplexing by coherent gain modulation (Akam and Kullmann, 2014), where a periodic drive to the decoding circuits may be used to separate the inputs arriving at different phases of the drive. We used theta oscillations to modulate the gain and amplify inputs arriving at some phases while attenuating the effect of others.

## Discussion

We showed that the MEC interneurons are receptive to external inputs only within cyclic windows defined by theta oscillations. Further, theta oscillations corralled stellate cells to spike synchronously at a phase where inhibitory interneurons were hyperpolarized and less receptive to excitatory inputs. This prevented stellate spikes from depolarizing randomly connected postsynaptic inhibitory interneurons that could compete with external inputs to other neurons. This mechanism to generate reliable sequences can also be harnessed to ensure that the EC selectively gates inputs such that some inputs that arrive at specific phases are transmitted to postsynaptic targets while others are blocked. Note that the sequences considered here are distinct from theta sequences (Foster and Wilson, 2007), where the neurons are aligned within a cycle of theta to form a compressed representation.

### Tuning properties of interneurons

**Figure 8.**
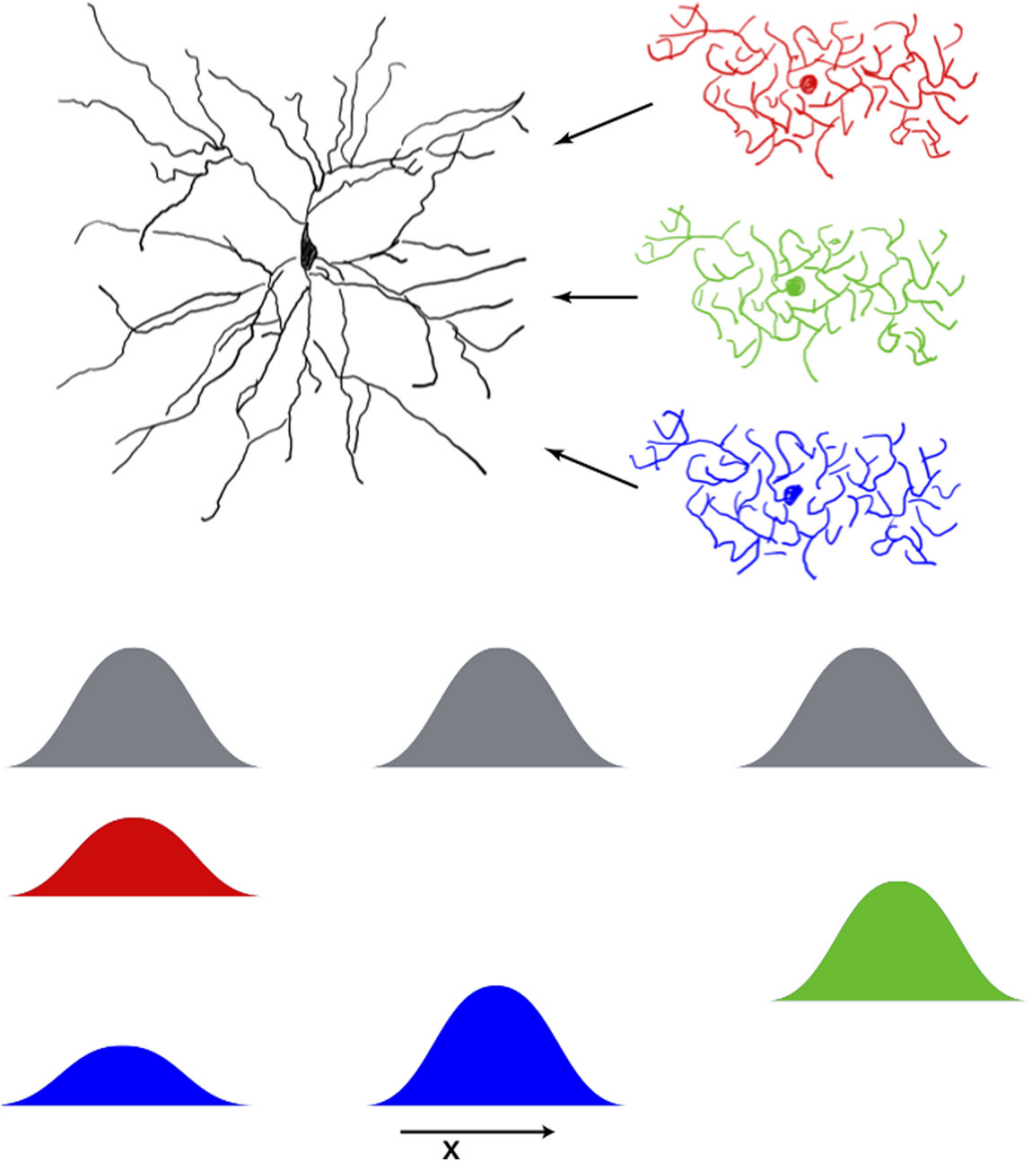
Aperiodic inputs to stellate cells. Stellate cell receives input from three inhibitory interneurons. The gray traces show the periodic receptive field of the stellate cell. The colored traces show the receptive fields of the inhibitory interneurons. The summed input from the inhibitory interneurons is periodic while individual interneurons possess broad and aperiodic spatial receptive fields.

Inhibitory interneurons in Layer II of the MEC are broadly tuned to spatial location and, unlike grid cells, do not show periodic firing fields (Buetfering et al., 2014). Subsequent studies have further emphasized that PV+ interneurons are necessary to form grid-like receptive fields. Selectively turning off PV+ interneurons eliminates grid-like receptive fields while preserving the receptive fields of other spatially tuned neurons. They found that the phases of grid cells providing input to a particular interneuron were uncorrelated and suggested that this may be why interneurons had broad and aperiodic firing fields. These observations present a challenge to attractor models in general that predict, hexagonally symmetric patterns of activity in the activity of grid cells would be reflected in the activity of inhibitory interneurons as well. We argue that these concerns may be addressed in our model (and in some attractor models) by requiring that the effective or summed inhibition to each grid cell must be spatially periodic, while allowing individual neurons to be broadly spatially tuned (Roudi and Moser, 2014). A plausible architecture where broadly tuned interneurons with aperiodic spatial receptive fields can provide spatially periodic input to stellate cells is shown in Figure 8. Each stellate cell receives inputs from a pool of inhibitory interneurons. Each neuron in this pool has a spatial receptive field that is centered around one or multiple peaks of the periodic receptive field of the stellate cell it innervates. While the receptive fields of each inhibitory interneuron shown in Figure 8 is non-periodic, the summed input of all the interneurons is periodic. Since our model depends on external inputs to drive the activity of inhibitory interneurons and stellate cells, this scheme can be easily implemented. In attractor models recurrent excitatory input must follow a local and uniform architecture. In contrast, in our model, each interneuron receives input from several randomly selected stellate cells, an architecture that appears to be closer to that described in Buetfering et al. (2014). Since these recurrent excitatory inputs to the interneurons are relegated to the resistant phase of the theta oscillations, they do not affect the response of the network to external drive.

### Stability in the absence of theta oscillations

**Figure 9.**
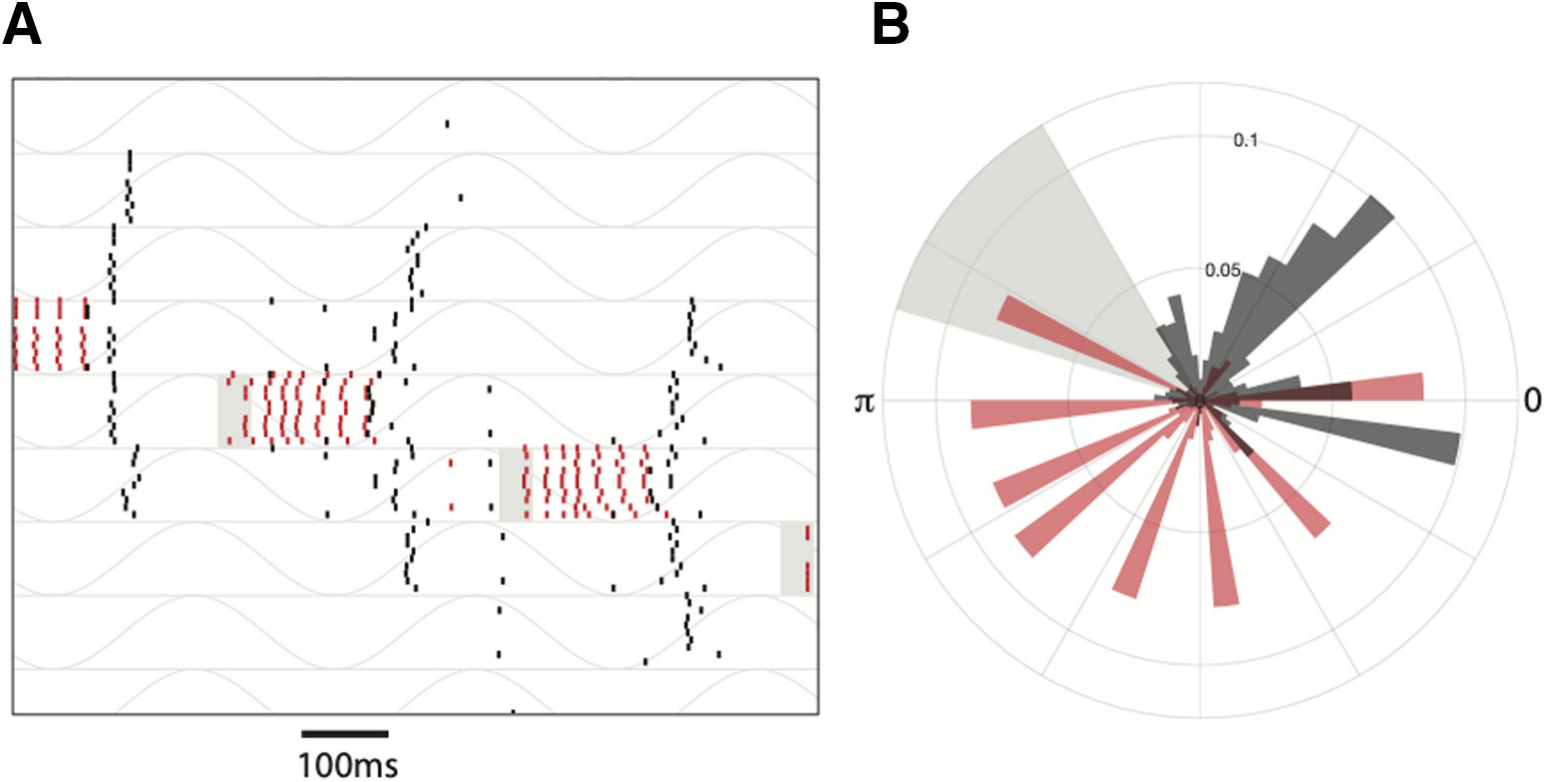
Response of the network to a slow oscillation (2 Hz). The input (shown in gray) toggles the activity of interneurons that continue to generate a burst of spikes (***A***). Rebound spikes by stellate cells occurred at a phase that did not perturb the sequence. ***B***, Histogram of the phase at which the stellate cells (dark bars) and the inhibitory interneurons (red bars) generate spikes.

Our model suggests that stable sequential activity is contingent on the presence of theta oscillations. In the absence of theta, multiple traversals over a given region of space failed to evoke a reliable response that is required to form grid-like receptive fields. However, recordings from Egyptian fruit bats show that grid-like receptive fields can be formed in the absence of continuous theta oscillations (Yartsev et al., 2011). This seems at odds with our model and experiments in rodent MEC where excising theta reversibly perturbs the grid-like structure of the receptive fields of MEC neurons (Koenig et al., 2011). One way to reconcile these contradictory observations is to assume that the MEC network in bats receives large amplitude inputs compared with smaller amplitude inputs in rodents. This can lead to a stable response even in the absence of theta oscillations (Fig. 4*B*, bottom traces). However, given that the MEC is a hub that receives multiple inputs, relying only on the amplitude of the input would impair its ability to selectively respond to some inputs while ignoring others that are equally salient. An additional layer of control can multiplex between similar inputs. Several lines of evidence suggest that theta oscillations might indeed be playing this role in rats. Despite its prominence in rodents, phase dependent gain modulation does not require a continuous fixed frequency oscillation. Interestingly, although bats lack a persistent oscillatory signal like theta in rats, they generate fluctuating low frequency local field potentials to which a significant proportion of principal cells are locked (Eliav et al., 2018). Can our model network use these low frequency inputs to generate a reliable output? We showed that reliability of our model network responses progressively deteriorates in the high theta frequency regime (>11 Hz; Fig. 6*B*). At low frequencies, the interneurons fire over a longer duration corresponding to a widened window of depolarization. The hyperpolarizing phase of the theta oscillation tends to shut the response of the interneurons and triggers a rebound excitation in stellate cells (Fig. 9*A*). Stellate cell spikes continue to occur during the hyperpolarizing phase of theta oscillations (Fig. 9*B*) and do not perturb the inhibitory network. Therefore, network responses to low frequency oscillations are reliable.

Bats show a large variability in the frequency of local field potential fluctuations unlike theta oscillations in rodents that vary over a smaller range. In our model network the input driven switch from one interneuron to another is rapid because of inhibitory competition between fast-spiking interneurons and occurs within a single theta cycle. Therefore, the network activity can respond to fluctuations in a cycle-by-cycle manner, relegating distractors to a hyperpolarized phase despite variations in instantaneous frequencies. Changes in instantaneous frequency that occur on a slower time scale than the switching time scale have little impact on perturbing the input driven dynamics of the network. Thus, the slow local field fluctuations seen in bats may be sufficient to evoke a reliable response in stellate cells.

### Role of neuromodulation in gating sequences

We showed that theta oscillations can gate the transmission of information between different brain regions. Mechanisms ranging from a cellular scale to that of populations of neurons can lead to coherent oscillations (Tiesinga and Sejnowski, 2009, 2010). One can selectively couple two regions by ensuring that the phase of theta is coherent across these regions and information is transmitted during a restricted phase window of theta. Interregional interactions via oscillatory phase coherence is not restricted to circuits including MEC, but widespread across many cortical and subcortical structures (Kay, 2005; Colgin, 2011, 2013; Kim et al., 2011; Liebe et al., 2012; Fries, 2015). A number of behaviors depend on recruiting a broad network of regions. For example, hippocampal and amygdalar circuits show theta coherence when animals are exposed to anxiety inducing situations (Adhikari et al., 2010). Working memory in rodents (Jones and Wilson, 2005) recruits hippocampal and medial prefrontal cortex via theta synchrony. Many adaptive behaviors coincide with an enhanced coherence in the phase of the local field oscillations between different brain regions (Reinhart et al., 2015; Tendler and Wagner, 2015). Memories at various stages of encoding, consolidation and retrieval invoke different configurations of brain regions that are dynamically assembled by theta phase coherence across these regions. During the early phases of learning an association between a conditioned and an unconditioned stimulus, the hippocampal, LEC coupling is characterized by phase synchronized theta oscillations. As learning progresses, the phase synchrony between the hippocampus and LEC decreases with a concomitant increase in LEC-medial prefrontal cortex synchrony (Takehara-Nishiuchi et al., 2012). Theta synchrony is a read-out of increased information transfer across brain regions.

However, finer control over the input during each theta cycle is required to ensure that MEC networks selectively listen to or ignore incoming inputs. What are the mechanisms that ensure the right inputs arrive at the right phase of theta? Inhibitory interneurons that participate in generating and maintaining hippocampal theta rhythms broadcast rhythmic inhibition that targets inhibitory interneurons in other areas (Gonzalez-Sulser et al., 2014). These, in turn, synchronize principal neurons. The effectiveness of inhibition onto principal neurons can alter the degree of synchronization and the phase of principal neuron spikes. In the hippocampal-medial prefrontal cortex circuit, this is likely controlled by neuromodulators like dopamine (Benchenane et al., 2010) that can effectively shift the phase of principal neuron spikes with respect to a theta oscillation and selectively couple it to brain regions downstream. Modulatory control can therefore create transient functional networks that flexibly serve different behavioral contingencies.

### Effects of synaptic plasticity on sequences

Our simulations operated in a regime where the system responded to a sequential external drive and moved the locus of activity from one neuron to another. Stellate cells spiked and registered the temporal location of this transition. In the MEC network, GABAergic connections onto the principal neurons show spike timing dependent plasticity that enhances the weights of inhibitory connections for those synapses where the postsynaptic stellate cell spikes after the inhibitory interneuron (Haas et al., 2006). Repeated sequential activation of the same network will therefore lead to changes in synaptic weight that introduce asymmetries in the network architecture (Mehta et al., 1997). Asymmetries become particularly relevant in the parameter regime where the network motif simulated in Figure 1*B* operates as an autonomous oscillator. Here, we simulated a simple network with two pairs of stellate cells and inhibitory interneurons that were symmetrically coupled. If this motif were extended to a chain of units with a directional asymmetry embedded in the chain, then the activity would propagate reliably along this asymmetry. These sequences may appear as episodes where the activity of the network is replayed (Ólafsdóttir et al., 2018) in the absence of any external inputs. Any brain region that is involved in flexible sequence learning must deal with a conflict between the intrinsic dynamics of the network, a consequence of asymmetries in the network that emerge from recent experiences, and a new sequential pattern representing novel experience. To learn the novel sequential pattern, the activity of the local circuits should be enslaved to the external input since many plasticity mechanisms are activity dependent. If local circuits are predisposed to dynamics dictated by internal asymmetries, the response to external input patterns would be perturbed by these internal dynamics. In the hippocampus a switch between initial encoding (listening to external input) and consolidation (or a reactivation across strengthened connections) is mediated by acetylcholine (Hasselmo et al., 1996; Káli and Dayan, 2000). In the MEC microcircuit, we propose that theta rhythmic inputs can effectively silence the intrinsic dynamics. In contrast, when external inputs are absent, intrinsic asymmetries take over and feed-forward excitation propagates reliable sequences along the network. The dynamics of the system is most likely a combination of driven and autonomous attractor-like dynamics. We speculate that the animal switches between using external cues to navigate and internal representations when external cues are absent or to consolidate memories of recently traversed trajectories.

In sum, our study highlights the central role of theta oscillations in generating reliable sequences and forming transient functionally connected networks. This is possible because of the fortuitous similarity between the time scales of theta oscillations and conductances of stellate cells together with the architecture of the MEC network.

*Note Added in Proof:* The codes and the documentation required to run the simulations and analyze the outputs are also available as the Extended Data 1.

10.1523/ENEURO.0059-20.2021.ed1 Extended Data 1 Theta gates reliable sequences Download Extended Data 1, ZIP file.

## Synthesis

Reviewing Editor: Upinder S. Bhalla, National Centre for Biological Sciences, Tata Institute of Fundamental Research; Decisions are customarily a result of the Reviewing Editor and the peer reviewers coming together and discussing their recommendations until a consensus is reached. When revisions are invited, a fact-based synthesis statement explaining their decision and outlining what is needed to prepare a revision will be listed below. The following reviewer(s) agreed to reveal their identity: Frances Skinner. Note: If this manuscript was transferred from JNeurosci and a decision was made to accept the manuscript without peer review, a brief statement to this effect will instead be what is listed below.

SYNTHESIS:

The reviewer and I concurred on the potential relevance of this study to understanding the role of theta oscillations in sequences. There are some essential things to clarify.

1. The authors seem to use a gradient of h-channel conductance along the dorsoventral axis as a key biological constraint in their model. However, it appears that the time-constant is the parameter which varies: J Neurosci. 2008 Sep 17;28(38):9414-25. doi: 10.1523/JNEUROSCI.3196-08.2008. Time constants of h current in layer ii stellate cells differ along the dorsal to ventral axis of medial entorhinal cortex Lisa M Giocomo, Michael E Hasselo In fact, the paper seems to explicitly rule out current amplitude changes (See Figure 3). Could the authors re-examine this assumption and show what it does to their model properties?

2. It would help to understand response stability by some analysis such as a PRC, to explain the required network properties.

In addition there are some useful minor comments to improve the MS.

REVIEWER’S COMMENTS

In this study, the authors examine computational models of MEC microcircuits to understand the stability and precision of sequential activities in the EC. The models rely on rebound spike characteristics in stellate cells (due to h-current) that 'match' theta, bringing about the importance of time-scales. It relies on connectivity (interneurons and stellate cells) and intrinsic properties, and the paper examines the link between theta oscillations and sequences in the MEC. Based on the authors' responses and my own reading of the manuscript, the modeling shows interesting observations of stability that relies on the interplay of theta and intrinsic/network properties.

My main comment/concern is in regard to the modeling choices and usage. First, the authors say many times about the changing h-channel conductance along the dorsoventral axis (Giocomo citation), but this is not the case. As far as I can tell from the relevant Giocomo papers, it is the activation time constant, not conductance, that varies along the axis (see cited 2008 JNeurosci Giocomo paper of the voltage clamp work showing this). Thus, the modeling showing changing output and bistability with h-conductance changes does not strictly apply here. Also see their response to Comment #4. This may not completely negate the overall results, but this needs to be rectified as to parameter choices/balances - I think they did not vary the intrinsic (stellate cell) properties within a given network model exploration?

Otherwise, while I think it is ok that they use modifications of other models (Rotstein et al and Wang and Buzsaki), it should be clear what the rationale and goal of doing so is. Here, it is about looking at the stability of the responses. It should be possible for the authors to actually understand this in some fashion by, for example, doing PRC on the stellate cell models to understand the cellular phase responses that underlies their network response. That is, one could potentially unpack their simulation observations, and explain the required 'balances' in the models for this to occur. All the various explorations (network connectivities etc.) are built on this (i.e., parameter 'balances' to get a rebound spike essentially), and so I think it is important to explore this in some fashion for the reader to understand and appreciate.

I note that Table 1 (unlike Table 2) is missing units. Also, the equation for mhs_infty is strange? I did not try to look at the code to check - is this a typo?

Minor

p.12 - misspelt ‘excitatory’

Reference ‘Wang (1996)’ is incomplete - Wang and Buzsaki etc.

p.19 red additions read more like Discussion points?

p.22 - Fig 5C should be 6c

Please expand caption for figure 6 - describe green tab for 6a. 6b does not have a yaxis label.
